# Supplementary material for: Association between oral microbiome and seven types of cancers in East Asian population: a two-sample Mendelian randomization analysis
Source: Front Mol Biosci. 2023 Nov 21;10:1327893. doi: 10.3389/fmolb.2023.1327893 (PMC10702768; doi:10.3389/fmolb.2023.1327893)
Supplement: Supplementary file 1 [file DataSheet1.ZIP › Supplementary Materials/MR plots for tongue/tongue═╝/Pancreatic cancer/╥╚╧┘░⌐_╔╕╤í╜ß╣√/pheno.2785_to_pancreatic cancer_leave_one_out.pdf]

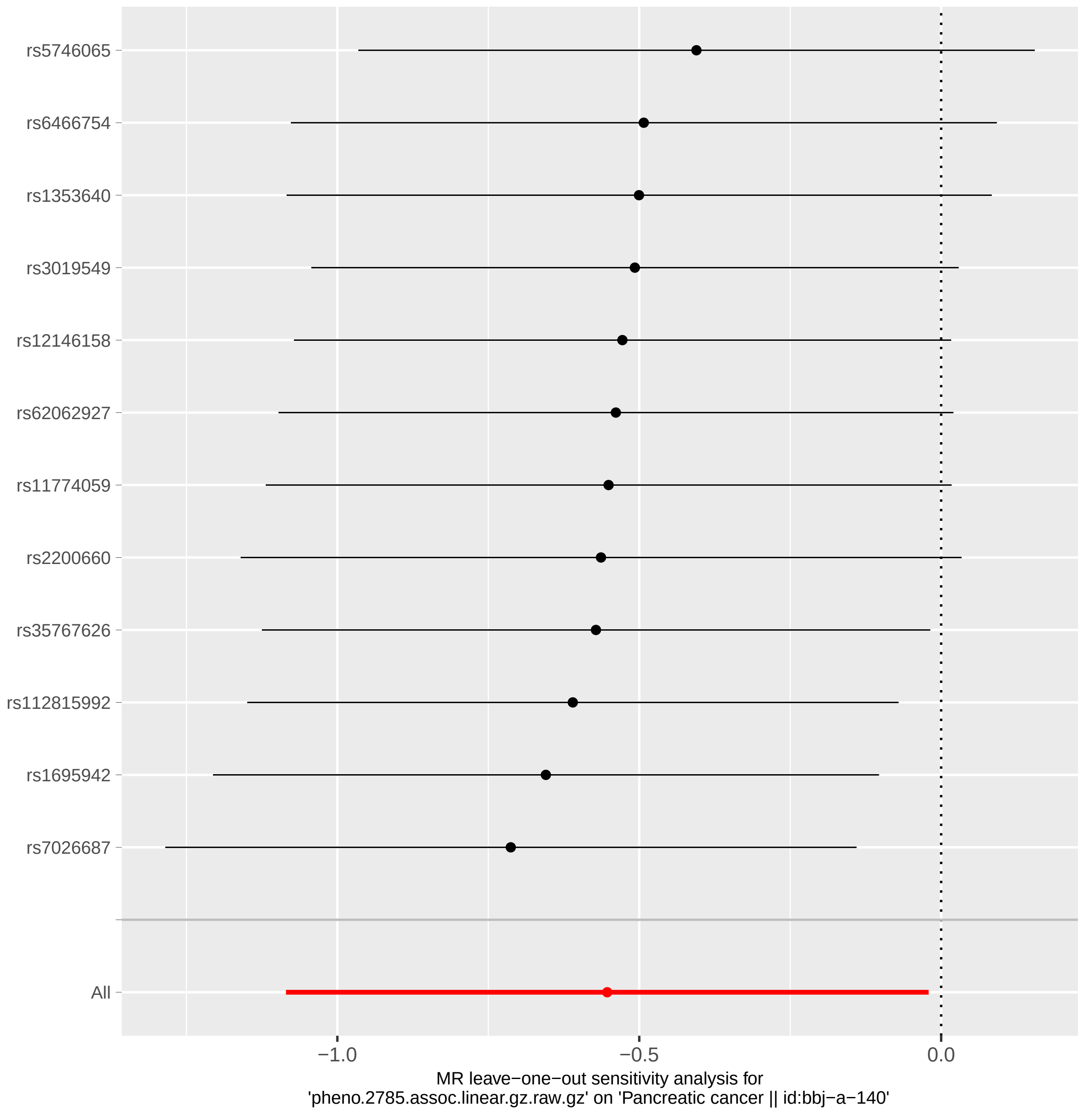
rs5746065
rs6466754
rs1353640
rs3019549
rs12146158
rs62062927
rs11774059
rs2200660
rs35767626
rs112815992
rs1695942
rs7026687
All
−1.0
−0.5
0.0
MR leave−one−out sensitivity analysis for
'pheno.2785.assoc.linear.gz.raw.gz' on 'Pancreatic cancer || id:bbj−a−140'
